# Language-assisted multimodal convolutional transformer pipeline for retinal lesions segmentation

**DOI:** 10.1038/s41598-026-55337-8

**Published:** 2026-06-11

**Authors:** Wilayat Khan, Mohammad Alsaffar, Muhammad Faisal Abrar, Taimur Hassan

**Affiliations:** 1 Department of Computer Engineering, University of Ha’il, Ha’il, Saudi Arabia; 2 Department of Information and Computer Science, University of Ha’il, Ha’il, Saudi Arabia; 3 Department of Software Engineering, University of Ha’il, Ha’il, Saudi Arabia; 4 Department of Applied Artificial Intelligence and Robotics, Aston University, Birmingham, UK

**Keywords:** Vision language models, Retinal lesions segmentation, Optical coherence tomography, Deep learning, Computational biology and bioinformatics, Diseases, Health care, Mathematics and computing, Medical research

## Abstract

Retinal lesion segmentation is one of the critical tasks to analyze retinal diseases. Many researchers have proposed deep-learning models to extract lesions from the retinal scans. However, these models often rely on image features that might not be clinically meaningful for identifying the retinal lesions. Additionally, these models need pixel-level ground truths, which are challenging to procure in the real world. To overcome these issues, we present a novel language-assisted multimodal convolutional transformer pipeline that aligns image features with the text features, where the text features are extracted from the prompts that contain clinically meaningful information about the retinal lesions, and the image features are generated from the retinal scans. This alignment between image and text features is established with one-time training using the proposed loss function. Afterward, the proposed network can robustly extract retinal lesions across different datasets at the inference stage. Moreover, since the proposed network infers learning from the text prompts, it does not require additional training rounds using pixel-level ground truth annotations to adapt to new datasets like the state-of-the-art methods. The proposed network is thoroughly tested on six public datasets, and it outperforms the state-of-the-art by achieving up to 7.77% improvements in terms of intersection-over-union.

## Introduction

Human vision is formed when light passes through the biconvex lens into the retina^1^. The retina is a thin membrane lined with special light-sensitive cells that convert the projection of an object represented by the light into electrical signals. These signals are further sent to the visual cortex of the brain for processing and interpretation of visual information^2,3^. The retina contains two prime regions: the macula, which provides central vision, and the peripheral region, responsible for side vision^4^. Retinal diseases, or retinopathy, encompasses a range of microvascular abnormalities in the retina associated with various medical conditions, such as autoimmune disorders^5,6^. However, it is most commonly linked to diabetes, where it is termed Diabetic Macular Edema (DME). DME is characterized by swallowed macula in optical coherence tomography (OCT) imagery, which usually leads to blurred and distorted vision^7^. The presence of lesions, such as hard exudates (HE), sub-retinal fluid (SRF), and intra-retinal fluid (IRF), are the prime signs that may represent DME, and they can be objectively visualized in OCT imagery in early stages^8^. Another common type of retinal disorder is age-related macular degeneration (AMD)^9^. In AMD subjects, the retinal lesions, such as drusen deposits of lipids and proteins and choroidal neovascularization (CNV) form beneath the retina, which can be effectively screened through OCT imagery. Moreover, AMD is further categorized into two forms, namely, the dry and the wet form. The early manifestation of drusen accumulation formed due to the degeneration of retinal pigment epithelium (RPE) indicates dry AMD. However, the progression of the disease produces CNV, which is the abnormal growth of the vascular proliferations that emanate from the choroid to reach the retina, leading also to the accumulation of retinal fluids. AMD, in this stage, is known as the wet AMD^10^.

Color fundus photography, fundus fluorescein angiography, and OCT are the diagnostic approaches to identify these diseases non-invasively. Among them, OCT scans are widely considered to be the most helpful modality in diagnosing various retinal diseases accurately. Retinal OCT imaging has emerged as a commonly used modality for diagnosing various kinds of retinal pathologies, including DME and AMD^8^. In the clinical setting, OCT is considered the best method to identify the formation of retinal lesions, such as HE, IRF, SRF, Drusen, and CNV, to screen DME and AMD pathologies^9^. Clinical practice has further explored the applicability of OCT imagery to objectively assess the formation of AMD and DME-related abnormalities as compared to other modalities^11^. Besides, clinically significant symptoms related to the diseases, such as retinal holes, retinal detachment, and age-related retinoschisis, have been found to be accurately revealed with the peripheral OCT imagery^12^.

### Motivation

Many researchers have developed automated systems to screen retinal diseases. These systems are based on imaging modalities involving fundus photography and OCT scans^10^. The recent studies also introduced attention mechanisms^13^ and hybrid convolutional networks^9^ for lesion-focused analysis of retinal diseases. However, most of these methods do not incorporate clinical insights into a lesions extraction or disease classification process and, therefore, are not well-suited for practical clinical applications for objectively assessing retinal diseases^14^. Furthermore, these methods require extensive ground truth annotations, which are challenging and infeasible to procure in a clinical setting to extract retinal lesions. Therefore, there is a dire requirement to develop deep learning systems that can incorporate clinical manifestations into a lesion extraction process to produce an accurate and objective segmentation of retinal lesions. Furthermore, as ground truth annotations are hard to procure in clinical settings, and they are also highly dependent on the scan characteristics^15^, so there is a need to develop deep learning models that should be able to leverage supervision from the one-time generated clinical prompts rather than ground truth annotations to extract retinal lesions from the OCT scans acquired with different machinery.

## Related work

Analysis of retinal images has been one of the most researched topics, where clinicians and researchers have different various machine learning techniques to detect different retinal lesions, which aid in screening retinal diseases using fundus^1^ and OCT scans^16^. However, clinically, OCT scans are considered much better than fundus photography since these are more sensitive to recognizing minor pathological abnormalities in the retina at an earlier stage^10^. This section presents a review of the recent methods that are designed to extract retinal lesions and screen retinal diseases.

### Machine learning methods

In the last few decades, many automated methods have been developed for performing retinal lesion extraction and disease classification tasks. The conventional machine learning approaches relied on handcrafted features that could extract retinal fluids^17^ or classify retinal diseases^16^. However, the use of subjective handcrafted features limits such approaches to work well on very few samples^18^. Deep learning models addressed this issue by significantly improving the performances of the existing methods by many folds towards extracting retinal lesions and classifying disease patterns^9^. Here, Fang et al.^19^ introduced the use of CNNs for segmenting the retinal layers from OCT images of non-neovascular AMD subjects. Further work introduced advanced attention mechanisms that aligned deep learning models to produce better identification of retinal abnormalities^15^. Activation maps and rule-based approaches have also been proposed in the literature to classify retinal diseases^13^. Moreover, Zang et al.^20^ employed deep learning and graph search techniques to segment the optic disc. Liu et al.^21^ devised a variation of the U-Net model, called MDAN-UNet, which utilizes multiscale attention to segment retinal lesions^21^. Sun et al.^22^ combined CNN with the traditional classifier to screen abnormal retinal pathologies. Li et al.^23^ devised a graph convolutional model to extract the retinal layers from the retinal scans. Butola et al.^24^ have proposed a lightweight model that has two convolutional layers and one fully connected layer for the classification of the OCT scans between AMD and DME categories. Further, Das et al.^25^ enhanced the resolution of OCT images to diagnose AMD better with the help of adversarial networks. Wang et al.^26^ proposed a reconstruction model to produce normal OCT scans.

Weakly supervised methods have, to some degree, alleviated the demand for extensive ground truth labeling, such as those reported in^27,28^. Unfortunately, most of these works face significant problems in achieving good performance toward extracting retinal lesions or screening retinal diseases^9,13,24,26,29^. Moreover, these methods require extensive ground truth annotations to learn lesions extraction and disease classification tasks across multi-vendor OCT scans, which are both impractical and infeasible to procure in real-world clinical settings^13,15^.

### Large language models

Many people have highlighted the benefit of using large language models (LLMs) to identify retinal disabilities within clinical pathways^15^. Huang et al.^30^ assessed LLMs to manage glaucoma and other retinal diseases. They used GPT-4^31^ to generate diagnostic responses against the standard clinical questions and compared the responses of GPT-4^31^ with the recommendations of expert clinicians. They found that LLMs can develop clinically significant responses against the standard clinical questions for managing the progression of retinal abnormalities, including glaucoma. Similarly, Kleinig et al.^32^ compared different LLMs in ophthalmology and concluded that GPT-4^31^ and Bing AI^33^ generate the most accurate and concise responses as compared to the rest of the model toward handling ophthalmic and retinal diseases. On the other hand, Ong et al.^34^ used LLMs to generate the International Classification of Diseases (ICD) codes from the text prompts related to retinal disabilities. They founded that LLMs like ChatGPT^35^ can ease the load of doctors by generating accurate ICD codes from the textual data. Apart from this, Khanian et al.^36^ used LLM models, such as ChatGPT^35^, and Google Bard^37^, to generate educational and easier-to-understand notes for the doctors about the retinal uveitis. They identified that ChatGPT^35^ can be used in hospitals to generate accurate educational material, especially related to uveitis.

The recent literature evidences that the LLMs possess capabilities to produce clinically meaningful prompts pertaining to retinal disorders. These prompts provide tremendous scope for researchers to use them in training machine learning models in the context of retina, as they facilitate precise screening for retinal abnormalities irrespective of the scanner types, and the datasets^30,32,34,36,38,39^.

## Contributions

This paper presents a novel language-driven multimodal convolutional transformer pipeline that aligns image and text features, where the text features are extracted from the prompts that contain clinically significant manifestations of the retinal lesions, and the image features are generated from the retinal OCT scans. This alignment between image and text features allows the proposed network to robustly segment retinal lesions depicted within the retinal OCT images of across different datasets without needing any ground truths. Moreover, this alignment between image and text features within the proposed network is performed in training phase using the $L_s$ loss function, which maximizes the distance between samples of different lesion classes while minimizing the distance between samples of the lesion class. Moreover, since the proposed network infers learning from the text prompts, it does not need additional ground truths in order to process new datasets like state-of-the-art (SOTA) models. The proposed model is tested across six public datasets where across each dataset, it outperforms SOTA methods in terms of mean intersection-over-union (mIoU), mean dice coefficient (mDC), and mask mean average precision ($\mu _{map}$) scores. Overall, the key contributions of this work are:

- A novel language-assisted multimodal convolutional transformer pipeline is proposed in this paper that can extract retinal lesions from the OCT scans affected by DME and AMD diseases.
- The proposed method leverages supervision from the clinical prompts rather than ground truth annotations to identify the presence of retinal lesions.
- Due to the ability of the proposed method to learn retinal lesions segmentation tasks using text prompts rather than image-based ground truth annotations. It enables the proposed method to produce remarkable segmentation results when it is adapted to different retinal OCT datasets with one-time training (see “Proposed method”, “Training stage” and “Inference phase” for more details).

The rest of the sections are organized as follows: “Proposed method” presents the proposed framework in detail. “Experimental setup” enlists experimental protocols. “‘Results” presents the results of the proposed framework and its comparison with the SOTA method. “Discussion” discusses the proposed scheme in detail, and “Conclusion” concludes the paper and envisages future directions.

**Fig. 1:**
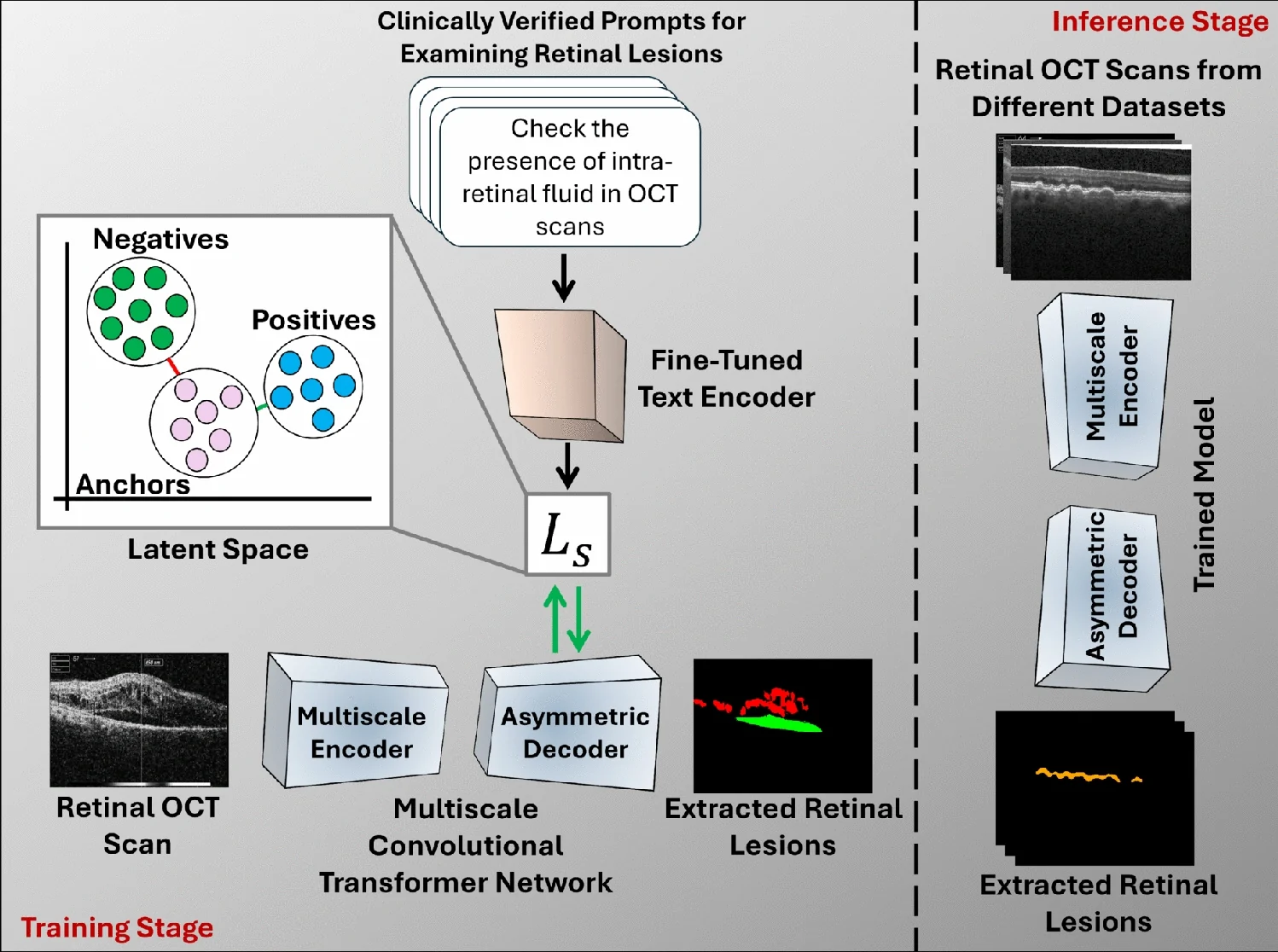
Overview of the proposed scheme. In the training phase, the proposed multiscale convolutional transformer network is penalized using $L_s$ loss function to extract lesions, such as HE, Drusen, IRF, SRF, and CNV, from the OCT scans. Here, the model is trained to extract by aligning its image features, generated from the OCT scans, in accordance with the text features, generated from text prompts that represent clinically significant manifestations of the underlying disease pattern. The text features are generated from the clinical prompts using the text encoder, which is pre-trained to identify retinal lesions associated with DME and AMD diseases as per the clinical standards. Since the proposed model is trained using the text features generated from the clinical prompts, it does not need any ground truths to learn pixel-level lesion segmentation tasks, and with one-time training using the $L_s$ loss function, the proposed model has the capabilities to process different datasets, containing OCT scans acquired from various machinery, to robustly extract retinal lesions at the inference stage, unlike SOTA methods.

## Proposed method

The block diagram for the proposed framework is shown in Fig. 1. We can observe that during the training stage, the proposed multiscale convolutional transformer model learns to extract retinal lesions, such as HE, IRF, SRF, Drusen, and CNV, from the OCT scans by aligning its feature embeddings with the text features using the $L_s$ loss function. These text features are produced from the text prompts using the text encoder where the text prompts represent clinically significant manifestations of the underlying disease pattern. The text encoder has been pre-trained to identify retinal lesions, such as HE, IRF, SRF, Drusen, and CNV, that are associated with AMD and DME pathologies and are also observed by the clinicians during the retinal examination. Since the proposed model is trained using the text features extracted out of the text prompts, it does not require any ground truth annotations to learn pixel-level lesion segmentation tasks, and with one-time training using the $L_s$ loss function, the proposed model possesses the capabilities to process different datasets, containing OCT scans acquired from various machinery, to robustly extract retinal lesions at the inference stage, unlike SOTA works. It is worth noting that the text encoder within the proposed system generates text features that correspond to pixel-level retinal lesion categories (not the class-level). Moreover, the $L_s$ function, as expressed in Eq. (3), tries to minimize the distance between text and image features belonging to the same lesion classes while maximizing the image and text features distance belonging to different lesion classes during the training stage. The image features are derived not from the image as a whole, but rather from the pixels of the candidate input image. Moreover, The detailed description of the proposed multiscale convolutional transformer network, as well as the training and inference stages, are explained in the subsequent sections.

### Multiscale convolutional transformer network

To extract the retinal lesions from the OCT scans, we propose a multiscale convolutional transformer network. The proposed model is composed of a multiscale encoder that is coupled with the asymmetric decoder. The encoder end of the model generates latent space representations from the given OCT scan by decomposing it into multiple scales and passing them through the series of convolutional, batch normalization (BN), rectified linear units (ReLU) layers coupled with the identity, and shape preserving blocks. The purpose of employing multiscale decomposition within the encoder is to enable it to extract distinct feature representations that encompass coarser to finer details of the retinal lesions contained within the candidate OCT scan. These multiscale features are then combined together and passed to the contextual refinement block, which further refines the features by retaining only the contextual information of the retinal lesions while suppressing the irrelevant details. The contextual insights about the retinal lesions are majorly retained within the latent features through the use of a hierarchical decomposition block (HDB), which is appended at the end of the contextual refinement block. Architecturally, the identity blocks (IB) are made of three convolutional layers, three batch normalization layers, and two ReLU activations connected sequentially. The residual connection adds the input kernels to the output of the cascaded layers and enables the network to capture distinct features for the identification of the retinal lesions. Shape-preserving blocks (SPB) consist of four convolutional layers, each followed by a BN and two ReLUs. Here, the residual connection combines the output of one convolution and one BN with the rest of the cascaded layers to retain the retinal lesions’ appearances during the feature decomposition process. Similarly, the HDB block comprises a parallel combination of average pooling with variable factors, BN, and ReLU layers, which are resized to a resolution of $18 \times 24 \times 512$ and are concatenated together to produce the rich feature representations to identify different retinal lesions.

Once the latent features are generated by the multiscale encoder, they are passed to the asymmetric decoder that contains a series of convolution, ReLU, batch normalization, and transformer blocks to extract the retinal lesions. Here, instead of using a conventional multi-head self-attention mechanism that is typically used in transformers^40^, we propose the use of hardswish activation^41^ as a token mixer to extract retinal lesions. The reason for using hardswish activation as a token mixer is because it produces remarkable segmentation results while drastically reducing the computational costs as compared to the multi-head self-attention mechanism^40,41^. Mathematically, hardswish activation function is defined in Eq. 1:

1 $$\begin{aligned} Hardswish(x) = {\left\{ \begin{array}{ll} 0 & x_{emb}\le -3 \\ x_{emb} & x_{emb}\ge +3 \\ \frac{x_{emb}.(x_{emb}+3)}{6} & otherwise \end{array}\right. }, \end{aligned}$$

where $x_{emb}$ denotes the normalized feature embeddings passed to the hardswish activation function. Moreover, the multilayer perceptron (MLP) at the end of the transformer unit consists of two linear layers and a Gaussian Error Linear Unit (GELU)^42^ activation (*σ (.)*), as defined in Eq. 2:

2 $$\begin{aligned} y_{emb}' = \sigma (y_{emb} W_{1} + \beta _1)W_{2} + \beta _2, \end{aligned}$$

where $y_{emb}$ represents the input embeddings to MLP, $y_{emb}'$ denotes the MLP output, $W_{1,2}$ are the learnable weights, and $\beta _{1,2}$ are the bias vectors within the MLP linear layers. The integration of a multiscale encoder and asymmetric decoder within the multiscale convolutional transformer network produces promising lesion segmentation results as compared to the SOTA models. Furthermore, we have tested the applicability of the proposed scheme with other SOTA segmentation models like SegFormer^43^ and SETR^44^. Although the proposed scheme works well with these models. However, the multiscale convolutional transformer network produces the best retinal lesion extraction performance as compared to these models (see “The Optimal Segmentation Model” for more details).

### Training stage

The proposed model undergoes a one-time training routine using $L_s$ loss function to learn retinal lesions extraction tasks from the retinal OCT scans. The $L_s$ loss function measures the deviation between the image features (generated by the multiscale convolutional transformer network from the given OCT scan) and the text features (produced from the clinical prompts using the pre-trained text encoder) in a latent space using Euclidean distance. Then, the $L_s$ loss function tries to minimize the Euclidean distance between anchors and positives while increasing the Euclidean distance between negatives and anchors within the latent space during each training epoch. Anchors are the image features produced from the given OCT image belonging to a positive class, while positives are the text features produced by the text encoder from the text prompts that represent a positive class. Moreover, negatives are the text features produced by the text encoder from the text prompts representing the non-positive class. Once the training is completed, the proposed multiscale convolutional transformer network can extract retinal lesions from the OCT scans affected by DME and AMD pathologies. Since the learning here is established using the clinical text prompts, which emphasizes identifying the clinically relevant information about the retinal lesions rather than their pixel-level representations contained within the OCT scans, the proposed model possesses the capability to extract retinal lesions from multi-vendor retinal OCT scans contained within different datasets that are acquired by different machinery without the need of going through extra re-training or fine-tuning rounds. So, with one-time training without requiring any hefty pixel-level ground truth annotations, the proposed model can perform retinal lesions extraction tasks using the retinal OCT scans of AMD and DME subjects at the inference stage, irrespective of the scanner characteristics. In subsequent sections, we discuss the pre-trained text encoder and the training aspects of the proposed network.

### Fine-tuned text encoder

The text encoder serves as a foundation for training the proposed model to extract retinal lesions from the AMD and DME-affected OCT scans during the one-time training round. Here, we first fine-tuned the text encoder offline on the two sets of clinical prompts generated, respectively, by the clinicians and the ChatGPT 4.0^31^. Afterward, we use it as a pre-trained model within the proposed scheme to generate text features which are then used to fine-tune the multiscale convolutional transformer network. The text encoder is trained offline for 100 epochs using ADAM optimizer with an initial learning rate of 0.01, batch size of 32, and categorical cross-entropy loss function. Both sets of prompts on which the text encoder was fine-tuned highlighted the clinical manifestations related to the retinal lesions that the doctors also observe in the OCT scans within the clinical setting. The prompts produced by ChatGPT 4.0^31^ are the augmented version of the prompts generated by the clinicians, and they were first cross-verified and validated by the panel of two expert ophthalmologists before we used them to train the text encoder. We noticed through ablation experiments, reported in “The optimal text encoder”, that combining both sets of prompts (generated by clinicians and ChatGPT 4.0^31^ yielded better-performing text encoder as compared to the variants that were trained on each set individually (see “The optimal text encoder” for more details).

### $L_{s}$ loss function

To train the proposed model for performing retinal lesions extraction task using the text features generated from the clinical prompts by the pre-trained text encoder, we designed $L_s$. $L_s$ is a linear combination of two objective functions, as expressed below:

3 $$\begin{aligned} L_s = \alpha _1 L_t + \alpha _2 L_{a}, \end{aligned}$$

4 $$\begin{aligned} L_t = \frac{1}{b_s}\sum _{i=0}^{b_s-1} max( \Vert {f(x_{a,i}) - f(x_{p,i})}\Vert ^2_2 - \Vert {f(x_{a,i}) - f(x_{n,i})}\Vert ^2_2 + \lambda , 0), \end{aligned}$$

5 $$\begin{aligned} L_{a} = -\frac{1}{b_s} \sum _{i=0}^{b_s-1} \log \left( \frac{ e^{-d(\cos (\theta _{i,a}), \cos (\theta _{i,p}))} }{ e^{-d(\cos (\theta _{i,a}), \cos (\theta _{i,p}))} + e^{-d(\cos (\theta _{i,a}), \cos (\theta _{i,n}))} } \right) , \end{aligned}$$

where $b_s$ denotes the batch size, *λ* denotes the distance margin, $f(x_a)$ denotes the features computed from the anchor samples, $f(x_p)$ denotes the features computed from positive samples, $f(x_n)$ denotes the features computed from the negative samples, $\Vert .\Vert ^2_2$ represents the L2-norm operator, $\alpha _{1,2}$ are the $L_s$ loss function hyperparameters which are empirically determined to be 0.5 to give equal contribution to the $L_t$ and $L_{a}$ functions while computing the $L_s$ loss. $\cos (\theta _{i,a})$ is an angular representation of anchors’ latent features, which is administered by the hyperparameter $\theta _{i,a}$. Moreover, $\theta _{i,a}$ is computed by taking the dot product between $f(x_a)$ and network weights *W*, i.e., $\theta _{i,a}=\arccos {\left( \frac{f(x_a).W}{|f(x_a)||W|} \right) }$. Similarly, $\cos (\theta _{i,n})$ and $\cos (\theta _{i,p})$ are the angular representation of the latent features extracted from the negative and positive samples, respectively, and $\theta _{i,n}$ and $\theta _{i,p}$ are computed in a similar manner as $\theta _{i,a}$. Apart from this, *d*(*u*, *v*) represents a Euclidean distance that is computed between the given *u* and *v* vectors.

### Inference phase

Once the proposed model is trained, it is applied to the test AMD and DME-affected OCT scans of six public different datasets to extract retinal lesions. Since the proposed model learns the lesions extraction tasks through the text features extracted from the text prompts rather than image-based ground truth annotations, it possesses an intrinsic ability to extract retinal lesions from scans belonging to different datasets without needing additional re-training or fine-tuning iterations. Therefore, with one-time training, the proposed model can be used to extract retinal lesions from the OCT images regardless of their scanner characteristics, the vendor artifacts, or the dataset variations. This adaptation ability of the proposed model makes it a very scalable and promising option to be deployed in the real hospital setting for extracting retinal lesions in a clinical setting, which can aid doctors in reducing their hefty workloads at a large scale, unlike SOTA lesions extraction models which require additional fine-tuning or re-training routines to work on scans with different scanner specifications^10,13^.

## Experimental setup

We elaborate in this section on the details of the datasets, implementation strategies, and metrics that were used to evaluate the proposed model and compare it with the SOTA methods.

### Datasets

For evaluating the proposed model against the SOTA works, we used six publicly available datasets. The overview of these datasets is presented below, and their summary is presented in Table 1. We also want to highlight that to evaluate the proposed model and to compare it with SOTA methods at the inference stage fairly, we used the pixel-level lesions annotations marked by the expert clinicians in^9^ for all of these datasets.

#### BIOMISA dataset

BIOMISA dataset^45^ contains a total of 4,163 OCT (1,064 AMD, 2,195 DME, and 904 normal) from 75 subjects (17 healthy, 31 DME, and 27 AMD). These scans were acquired using a Topcon 3D OCT-2000 scanner. Moreover, the retinal lesions within the BIOMISA dataset scans are marked at pixel-level by the expert clinicians^45^. Apart from this, we used the BIOMISA^45^ dataset in this work to train the proposed model one-time using its 80% scans, whereas the rest of the 20% scans from the BIOMISA^45^ dataset were used for the evaluation purposes at the inference stage.

#### Duke-1 dataset

Duke-1 dataset^46^ is one of the oldest retinal OCT datasets that contains a total of 38,400 scans, from which 26,900 scans reflect AMD and 11,500 scans show controlled (healthy) pathology. These scans were acquired using a Bioptigen OCT scanner. The AMD-affected scans within Duke-1^46^ contain the presence of Drusen, which are marked extensively by the expert clinicians in^9^. Moreover, in this research, we used the Duke-1 dataset^46^ to evaluate the proposed network at the test stage once it was one-time trained on the BIOMISA^45^ dataset.

#### Duke-2 dataset

Duke-2 dataset^17^ contains a total of 610 OCT images from 10 severe DME-affected subjects. These scans were acquired using a Spectralis Heidelberg OCT scanner. Moreover, the dataset comprises highly detailed markings for retinal layers and lesions, such as IRF, SRF, and HE, marked by expert clinicians in^9^. Apart from this, in this research, we used the Duke-2 dataset^17^ to evaluate the proposed network at the test stage once it was one-time trained on the BIOMISA^45^ dataset.

#### Duke-3 dataset

Duke-3 dataset^47^ contains a total of 723 scans that represent AMD, 1,101 scans that represent DME, and 1,407 scans that show healthy pathology. These scans were acquired using a Spectralis Heidelberg OCT scanner. Moreover, the scans within Duke-3^47^ contain retinal lesions, such as SRF, IRF, HE, Drusen, and CNV, which are marked extensively by the expert clinicians in^9^. Apart from this, in this research, we used the Duke-3 dataset^47^ to evaluate the proposed network at the test stage once it was one-time trained on the BIOMISA^45^ dataset.

#### Rabbani dataset

Rabbani^48^ contains a total of 4,241 OCT scans, which are acquired from 50 normal, 48 AMD, and 50 DME-affected subjects. These scans were acquired using a Spectralis Heidelberg OCT scanner. Moreover, the scans within the Rabbani dataset^48^ contain retinal lesions, such as SRF, IRF, HE, and Drusen, which are marked extensively by the expert clinicians in^9^. Apart from this, in this research, we used all the scans of the Rabbani dataset^48^ for evaluating the proposed scheme at the testing stage once it is one-time trained on the BIOMISA^45^ dataset.

#### Zhang dataset

Zhang dataset^29^ contains 109,309 OCT images representing healthy, wet AMD (CNV), dry AMD (Drusen), and DME pathologies. These scans were acquired using a Spectralis Heidelberg OCT scanner. Moreover, the scans within Zhang^29^ contain retinal lesions, such as SRF, IRF, HE, Drusen, and CNV, which are marked extensively by the expert clinicians in^9^. Apart from this, in this research, we used all of the 109,309 OCT scans belonging to the Zhang dataset^29^ to evaluate the proposed network at the test stage once it was one-time trained on the BIOMISA^45^ dataset.

**Table 1:** A detailed description of six datasets along with the training-test pairs that are used in this work. ‘–’ indicates the samples from the given dataset were not used for training at all. *CNV* choroidal neovascularization, *IRF* intra-retinal fluid, *SRF* sub-retinal fluid, *HE* stands for hard exudates.

Dataset	Training	Testing	Pathologies	Marked Lesions
BIOMISA^45^	3330	833	AMD	Drusen, CNV
			DME	IRF, SRF, HE
			Normal	
Duke-1^46^	–	38,400	AMD	Drusen
			Controlled (Normal)	
Duke-2^17^	–	610	DME	IRF, SRF, HE
Duke-3^47^	–	3,231	AMD	Drusen
			DME	IRF, SRF, HE
			Normal	
Rabbani^48^	–	4241	AMD	Drusen
			DME	IRF, SRF, HE
			Normal	
Zhang^29^	–	109,309	AMD	Drusen, CNV
			DME	IRF, SRF, HE
			Normal	

### Implementation protocols

The proposed scheme is implemented using Python 3.7.9 and TensorFlow 2.2.0 on Anaconda. Experiments were carried out on a machine with 128 GB of RAM, Core i9-10940 at 3.30 GHz CPU, with NVIDIA Quadro RTX 6000 GPU. First, we fine-tuned the text encoder offline for 100 epochs using batch size 32, ADAM with an initial learning rate of 0.01, batch size of 32, and categorical cross-entropy loss function. Then, we use it as a pre-trained model to train the proposed multiscale convolutional transformer model on the BIOMISA^45^ dataset using the $L_s$ loss function. This one-time training routine lasted for 200 epochs using ADAM optimizer with an initial learning rate of 0.01 and batch size of 32 (To facilitate the research community, the source code is released along with complete documentation and the trained models at https://github.com/taimurhassan/lang_transformer).

### Evaluation metrics

The proposed system is assessed using standard segmentation metrics, such as mean intersection-over-union (mIoU), mean dice coefficient (mDC), and mask mean average precision ($\mu _{map}$). Evaluation of the proposed system with these metrics allows its fair comparison with SOTA methods across each dataset.

## Results

We give a detailed analysis on the performance of the proposed scheme in this section as well as the comparison of the proposed system with SOTA works across all six public datasets. Before presenting the comparative studies, we report the series of ablation experiments through which we determined the optimal backbones to be used within the proposed scheme.

### Ablation studies

We performed a series of ablation experiments to determine the: (1) optimal set of text prompts, (2) optimal text encoder, (3) optimal segmentation model, and (4) to compare the performance of the proposed model when it is trained using text prompt features versus combined text prompt features and pixel-level ground truths. The optimal text prompts gives wide exposure to the text encoder when it is fine-tuned offline to generate a set of discriminating features. The optimal text encoder generates a set of distinct text features that are used to train the proposed model to segment retinal lesions from the OCT scans. The optimal segmentation model allows the accurate extraction of retinal lesions across different datasets once it is trained.

#### The optimal set of text prompts

In the first series of ablation experiments, we determined the optimal set of clinical prompts through which we trained the text encoder to produce good text features that enable the proposed multiscale convolutional transformer model to accurately identify retinal lesions, such as HE, IRF, SRF, CNV, and Drusen, from the given OCT scans honoring the clinical standards. For this purpose, we first fine-tuned the text encoder (BERT^49^) on the text prompts generated by two expert clinicians. The total number of these clinical prompts was 1,000. Once the text encoder was fine-tuned on these prompts, we used it to train the multiscale convolutional transformer model to extract retinal lesions from the OCT scans of different datasets. Similarly, in another experiment, we used these 1,000 clinical prompts to generate a set of 10,000 prompts using ChatGPT 4.0^31^ and used these synthetic GPT-4 prompts to fine-tune the BERT^49^. In the third experiment, we combined the synthetic GPT-4 prompts as well as the original clinical prompts to fine-tune BERT^49^ to produce good text features to accurately train the proposed model to segment different retinal lesions from the OCT scans.

The performance of the proposed model to extract retinal lesions across different datasets using these three set of prompts is reported in Table 2. From Table 2, we can observe that when the text encoder (BERT^49^) was fine-tuned using the combination of clinical and synthetic GPT-4^31^ prompts, it led the proposed model to produce the best retinal lesions extraction performance in terms of mIoU, mDC, and $\mu _{map}$ scores across each dataset. This performance boost is achieved because the combined clinical and synthetic GPT-4^31^ prompts were able to give a broad exposure to BERT[49] to generate distinct features that encompass the detailed retinal lesions representations which are observed in the clinical practice. Moreover, the clinical prompts alone were not able to produce the optimal segmentation performance because they were limited in size, i.e., there were only 1,000 clinical prompts that expert clinicians produced. Manual generation of such a large number of prompts is impracticable for clinicians; hence, the use of ChatGPT 4.0^31^ for generating synthetic prompts and combining them with the original clinical prompts was able to produce the optimal segmentation performance of the proposed model across each dataset. These observations are also in line with the findings of the previous studies^30,32,38^, which indicate that ChatGPT 4.0^31^ has the potential to produce clinically relevant prompts for finding abnormal retinal pathologies^34,36,39^.

**Table 2:** Determining the optimal set of text prompts that are used in fine-tuning the BERT^49^. The optimal text encoder then generates the distinct text features that enable the proposed multiscale convolutional transformer model to produce the best retinal lesions segmentation performance. Bold indicates the best performance, while the second-best scores are in italics.

Dataset	Prompt	mIoU	$\mu _{map}$	mDC
Zhang^29^	Combined	**0.9025**	**0.8702**	**0.9487**
	GPT-4^31^	*0.8826*	*0.8449*	*0.9376*
	Clinicians	0.8579	0.8176	0.9235
BIOMISA^45^	Combined	**0.9489**	**0.9072**	**0.9737**
	GPT-4^31^	*0.9256*	*0.8832*	*0.9613*
	Clinicians	0.9061	0.8705	0.9507
Duke-1^46^	Combined	**0.9305**	**0.8961**	**0.9640**
	GPT-4^31^	*0.9147*	*0.8718*	*0.9554*
	Clinicians	0.8815	0.8486	0.9370
Duke-2^17^	Combined	**0.9398**	**0.8876**	**0.9689**
	GPT-4^31^	*0.9143*	*0.8683*	*0.9552*
	Clinicians	0.8868	0.8519	0.9400
Duke-3^47^	Combined	**0.9246**	**0.8823**	**0.9608**
	GPT-4^31^	*0.9053*	*0.8638*	*0.9503*
	Clinicians	0.8742	0.8352	0.9328
Rabbani^48^	Combined	**0.9154**	**0.8748**	**0.9558**
	GPT-4^31^	*0.9019*	*0.8601*	*0.9484*
	Clinicians	0.8837	0.8557	0.9382

#### The optimal text encoder

After determining the optimal set of prompts, we performed another series of experiments to determine the optimal text encoder, which can generate the distinct feature representation of the retinal lesions as per the clinical standards. These features then enable the proposed multiscale convolutional transformer model to segment retinal lesions from the OCT images of six public datasets with one-time training. We want to highlight here that within the proposed system, the inclusion of the text encoder is essential as it generates the text features from the text prompts, where these text features are used in training the multiscale convolutional transformer model to perform retinal lesions segmentation tasks. To determine the best text encoder, we fine-tune different models, such as BERT^49^, Word2Vec^50^, and XLNet^51^ using the combined clinical and synthetic GPT-4^31^ text prompts. Once these encoders were fine-tuned, we integrated them within the proposed scheme to train the proposed model toward extracting retinal lesions, such as HE, IRF, SRF, Drusen, and CNV, from the retinal OCT scans. Furthermore, we compared the performance of these text encoders with the classical schemes, such as one-hot encoding^52^, to generate text features.

The proposed model’s performance in extracting retinal lesions from the retinal OCT images when it was trained using the features produced by different text encoders is shown in Table 3. From Table 3, we can observe that the multiscale convolutional transformer network obtained the best lesion segmentation performance for each dataset when it is trained with the text features that are produced using BERT^49^. For example, with BERT^49^, the proposed model produces 5.41%, 9.14%, 8.28%, 7.92%, 7.50%, and 7.43% better results in terms of mean IoU compared to XLNet^51^, the second-best model, across Zhang^29^, BIOMISA^45^, Duke-1^46^, Duke-2^17^, Duke-3^47^, and Rabbani^48^ datasets, respectively. Since BERT^49^ led to the best retinal lesion segmentation performance across each dataset, we chose it as an optimal text encoder within the proposed scheme for the rest of the experiments.

**Table 3:** Determining the optimal text encoder across each dataset. Bold indicates the best performance, while the second-best scores are in italics.

Dataset	Encoder	mIoU	$\mu _{map}$	mDC
Zhang^29^	BERT^49^	**0.9025**	**0.8702**	**0.9487**
	Word2Vec^50^	0.7789	0.7228	0.8757
	XLNet^51^	*0.8536*	*0.8125*	*0.9210*
	One-Hot Encoding^52^	0.6307	0.5869	0.7735
BIOMISA^45^	BERT^49^	**0.9489**	**0.9072**	**0.9737**
	Word2Vec^50^	0.8201	0.7914	0.9011
	XLNet^51^	*0.8621*	*0.8249*	*0.9259*
	One-Hot Encoding^52^	0.7203	0.6718	0.8374
Duke-1^46^	BERT^49^	**0.9305**	**0.8961**	**0.9639**
	Word2Vec^50^	0.8075	0.7708	0.8934
	XLNet^51^	*0.8534*	*0.8194*	*0.9209*
	One-Hot Encoding^52^	0.7043	0.6651	0.8265
Duke-2^17^	BERT^49^	**0.9398**	**0.8976**	**0.9689**
	Word2Vec^50^	0.8069	0.7872	0.8931
	XLNet^51^	*0.8653*	*0.8241*	*0.9277*
	One-Hot Encoding^52^	0.6959	0.6489	0.8207
Duke-3^47^	BERT^49^	**0.9246**	**0.8823**	**0.9608**
	Word2Vec^50^	0.7977	0.7452	0.8874
	XLNet^51^	*0.8552*	*0.8123*	*0.9219*
	One-Hot Encoding^52^	0.6748	0.6217	0.8058
Rabbani^48^	BERT^49^	**0.9174**	**0.8748**	**0.9569**
	Word2Vec^50^	0.7879	0.7365	0.8813
	XLNet^51^	*0.8492*	*0.8025*	*0.9184*
	One-Hot Encoding^52^	0.6536	0.6035	0.7905

#### The optimal segmentation model

The third ablation experiment focused on identifying the best segmentation network that can be used within the proposed scheme to produce the best retinal lesion segmentation. For this purpose, we employed SOTA segmentation models, such as SegFormer^43^, and SETR^44^, along with the proposed multiscale convolutional transformer model within the proposed scheme, where all of these models were trained one-time using the training scans of the BIOMISA^45^ dataset, the text features produced by BERT^49^, and the $L_s$ loss function. The retinal lesions segmentation performance of these models across each dataset is reported in Table 4. From Table 4, we can notice that all three models were able to produce promising performance in extracting retinal lesions. These results signify the ability of the proposed scheme to use different segmentation models as a backbone. However, the proposed model, due to its novel architectural composition, was able to produce the best segmentation performance as compared to SegFormer^43^ and SETR^44^ across each dataset. Due to these performance improvements, we used the proposed model as an optimal segmentation backbone within the proposed scheme in the rest of the experiments.

**Table 4:** Determining the optimal segmentation model that produces the best retinal lesions extraction across each dataset when it is used within the proposed scheme. Bold indicates the best performance, while the second-best scores in ilalics.

Dataset	Encoder	mIoU	$\mu _{map}$	mDC
Zhang^29^	Proposed	**0.9025**	**0.8702**	**0.9487**
	SegFormer^43^	*0.8716*	*0.8391*	*0.9313*
	SETR^44^	0.8586	0.8145	0.9239
BIOMISA^45^	Proposed	**0.9489**	**0.9072**	**0.9737**
	SegFormer^43^	*0.9058*	*0.8576*	*0.9505*
	SETR^44^	0.8964	0.8328	0.9453
Duke-1^46^	Proposed	**0.9305**	**0.8961**	**0.9639**
	SegFormer^43^	*0.8824*	*0.8263*	*0.9375*
	SETR^44^	0.8649	0.8107	0.9275
Duke-2^17^	Proposed	**0.9398**	**0.8976**	**0.9689**
	SegFormer^43^	*0.8952*	*0.8286*	*0.9447*
	SETR^44^	0.8536	0.8104	0.9210
Duke-3^47^	Proposed	**0.9246**	**0.8823**	**0.9608**
	SegFormer^43^	*0.8629*	*0.8078*	*0.9264*
	SETR^44^	0.8343	0.7951	0.9096
Rabbani^48^	Proposed	**0.9174**	**0.8748**	**0.9569**
	SegFormer^43^	*0.8863*	*0.8426*	*0.9397*
	SETR^44^	0.8591	0.8027	0.9242

#### Text prompt and pixel-level supervision

In this series of ablation experiments, we constrain the proposed model using the dice loss as well, in addition to the proposed loss function using Eq. 5. The dice loss was computed using a limited number of pixel-level ground truth annotations and only for 10% of the scans from the BIOMISA dataset^45^. The results for this experiment are reported in Table 5. We can observe here that adding the ground truth supervision improves the segmentation performance on the BIOMISA dataset^45^. However, when the proposed model is applied to other datasets, the segmentation performance is degraded, and this is natural because the ground truth supervision is vulnerable to scanner variations, which are expected in different datasets. Due to the fact that the proposed scheme infers supervision from the text prompt features, it is able to produce reasonably decent segmentation performance across different datasets with one-time training and without requiring additional fine-tuning rounds. Furthermore, the proposed scheme eliminates the need for ground truth annotations, which was one of the major limitations of deploying deep learning models in clinical settings.

**Table 5:** Performance comparison between proposed model variants when it is trained using text prompt features only versus combined text + few pixel-level ground truth labels. Bold indicates the best performance across each dataset.

Dataset	Variant	mIoU	mDC
BIOMISA^45^	Text prompt supervision only	0.9489	0.9737
	Text prompt + pixel-level supervision	**0.9572**	**0.9781**
Zhang^29^	Text prompt supervision only	**0.9025**	**0.9487**
	Text prompt + pixel-level supervision	0.8831	0.9379
Duke-1^46^	Text prompt supervision only	**0.9305**	**0.9639**
	Text prompt + pixel-level supervision	0.9042	0.9497
Duke-2^17^	Text prompt supervision only	**0.9398**	**0.9689**
	Text prompt + pixel-level supervision	0.9124	0.9542
Duke-3^47^	Text prompt supervision only	**0.9246**	**0.9608**
	Text prompt + pixel-level supervision	0.9093	0.9525
Rabbani^48^	Text prompt supervision only	**0.9174**	**0.9569**
	Text prompt + pixel-level supervision	0.8923	0.9431

### Comparative studies

In the first series of comparative studies, we assessed the segmentation performance of the proposed model and compared it with the SOTA retinal lesions extraction models, such as Swin-UNETR^53^, HyFormer^54^, RLSRC^55^, and RAG-FW^9^, using mIoU, $\mu _{map}$, and mDC scores. Afterward, in the second series of experiments, we assessed the computational complexity and run-time efficiency of the proposed network and compared it with the SOTA works. Here, for fair comparison, we trained all the networks only once using the training and implementation protocols described in “Training stage”, and “Implementation protocols” using the training scans of the BIOMISA^45^, as reported in Table 1. The performance comparison of the proposed model with SOTA models in terms of mIoU, $\mu _{map}$, and mDC scores is reported in Table 6. From Table 6, we can observe that the proposed model was able to outperform the SOTA models across each dataset. For example, on the BIOMISA dataset^45^, the proposed model produced 4.34%, 5.54%, and 2.26% performance improvements over the second-best model in terms of mIoU, $\mu _{map}$, and mDC, respectively. Similarly, the proposed system obtained 4.01%, 5.29%, and 2.15% improvements on Zhang dataset^29^ in terms of mIoU, $\mu _{map}$, and mDC, respectively, 4.76%, 5.39%, and 2.52% improvements on Duke-1 dataset^46^ in terms of mIoU, $\mu _{map}$, and mDC, respectively, 5.50%, 5.06%, and 2.91% improvements on Duke-2 dataset^17^ in terms of mIoU, $\mu _{map}$, and mDC, respectively, 7.27%, 6.30%, and 3.92% improvements on Duke-3 dataset^47^ in terms of mIoU, $\mu _{map}$, and mDC, respectively, 7.77%, 5.70%, and 4.21% better results on Rabbani dataset^48^ in terms of mIoU, $\mu _{map}$, and mDC, respectively, as compared to the SOTA models. Similarly, from Table 7, we can observe that the proposed model requires 21.70% lesser GFLOPS and produces 11.22% better run-time performance in terms of milliseconds (ms) as compared to the SOTA models. Also, by observing the performance of the proposed model in Tables 6 and 7, we can notice that the proposed model provides the best trade-off between the segmentation results, computational complexity, and the run-time performance as compared to the SOTA retinal lesions extraction performance. These performance improvements stem from the novel architectural composition of the proposed model based on SPB, HDB, and IB blocks, along with the Hardswish activation mechanism within the transformer block of the proposed model. The integration of these blocks within the proposed scheme allows it to provide the best retinal lesion extraction performance across different datasets.

We also want to highlight that the retinal lesion segmentation performance of both the proposed system and state-of-the-art methods is relatively high across the Zhang^29^, BIOMISA^45^, Duke-1^46^, Duke-2^17^, Duke-3^47^, and Rabbani^48^ datasets, as reported in Table 6. This is primarily because retinal lesion segmentation in OCT imagery is comparatively less ambiguous than in fundus imagery, where challenges such as low contrast, structural overlap, and illumination variations make the task more difficult^56^. Furthermore, we would like to emphasize that the primary motivation of this work is not solely to outperform existing state-of-the-art methods. Instead, it is to develop a scalable, accurate, and robust retinal lesion segmentation framework that requires only one-time training and can generalize across OCT scans acquired from different scanners. This is achieved through the proposed pipeline’s ability to learn from clinically relevant text prompts rather than relying on scan-specific ground truth annotations.

**Table 6:** Comparison of the proposed scheme with SOTA approaches to segment retinal lesions. The best scores are in bold, while the second-best scores are in italics.

Dataset	Model	mIoU	$\mu _{map}$	mDC
BIOMISA^45^	Proposed	**0.9489**	**0.9072**	**0.9737**
	Swin-UNETR^53^	*0.9077*	*0.8569*	*0.9516*
	HyFormer^54^	0.8726	0.8336	0.9319
	RLSRC^55^	0.8453	0.8024	0.9161
	RAG-FW^9^	0.8542	0.8147	0.9213
Zhang^29^	Proposed	**0.9025**	**0.8702**	**0.9487**
	Swin-UNETR^53^	*0.8663*	*0.8241*	*0.9283*
	HyFormer^54^	0.8459	0.8126	0.9165
	RLSRC^55^	0.8075	0.7635	0.8934
	RAG-FW^9^	0.8118	0.7703	0.8961
Duke-1^46^	Proposed	**0.9305**	**0.8961**	**0.9639**
	Swin-UNETR^53^	*0.8862*	*0.8478*	*0.9396*
	HyFormer^54^	0.8715	0.8203	0.9313
	RLSRC^55^	0.8342	0.7862	0.9096
	RAG-FW^9^	0.8463	0.7984	0.9167
Duke-2^17^	Proposed	**0.9398**	**0.8976**	**0.9689**
	Swin-UNETR^53^	*0.8881*	*0.8521*	*0.9407*
	HyFormer^54^	0.8672	0.8169	0.9288
	RLSRC^55^	0.8249	0.7834	0.9040
	RAG-FW^9^	0.8436	0.8042	0.9151
Duke-3^47^	Proposed	**0.9246**	**0.8823**	**0.9608**
	Swin-UNETR^53^	*0.8573*	*0.8267*	*0.9231*
	HyFormer^54^	0.8439	0.8045	0.9153
	RLSRC^55^	0.8186	0.7768	0.9002
	RAG-FW^9^	0.8354	0.7904	0.9103
Rabbani^48^	Proposed	**0.9174**	**0.8748**	**0.9569**
	Swin-UNETR^53^	*0.8461*	*0.8249*	*0.9166*
	HyFormer^54^	0.8257	0.7943	0.9045
	RLSRC^55^	0.7906	0.7562	0.8830
	RAG-FW^9^	0.8083	0.7681	0.8939

### Qualitative evaluations

**Fig. 2:**
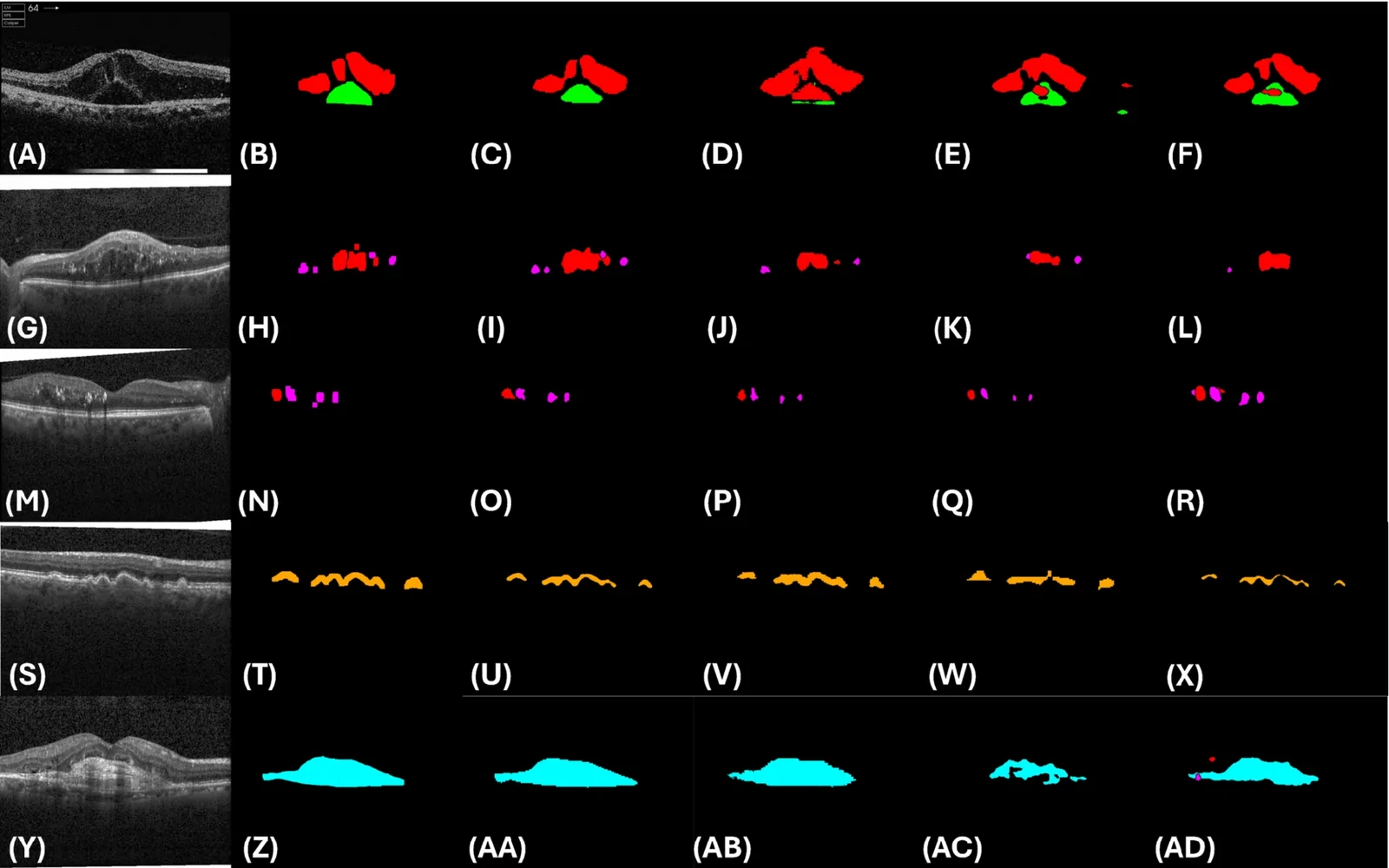
Visual examples showcasing the segmentation performance of the proposed network and the SOTA models. The first column showcases the exemplar scans taken from different OCT datasets reported in Table 1. The second column presents the annotations marked by the clinicians, while the third column displays the proposed model’s results. The fourth, fifth, and sixth columns show the results of Swin-UNETR^53^, RLSRC^55^, and HyFormer^54^, respectively. Additionally, the color scheme used in the visualizations is as follows: green represents SRF, cyan represents CNV, red represents IRF, magenta represents HE, and brown represents Drusen.

**Fig. 3:**
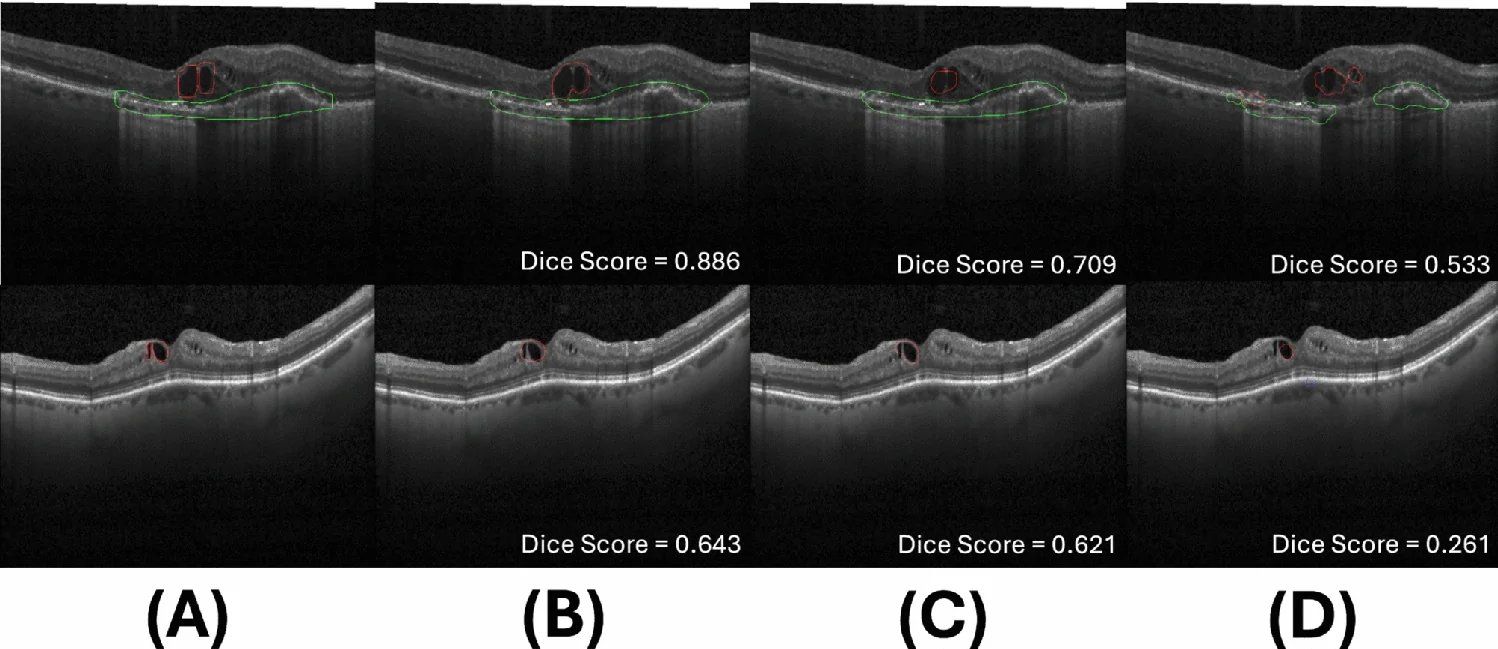
Visual examples demonstrating the retinal lesions extraction performance of the proposed system as compared to the ground truth and other works. (**A**) The ground truth, (**B**) shows the results of the proposed system, (**C**) shows the results of the Swin-UNETR^53^, (**D**) shows the results of the RLSRC^55^. Here, the masks are overlaid on the original images, and the dice score for each example is also reported.

We present in this section a detailed visual assessment of the multiscale convolutional transformer model for extracting retinal lesions from the OCT scans of six public datasets compared to the SOTA works. The qualitative assessment of the proposed network and the SOTA works is presented in Figs. 2 (A-AD), and 3. Here, we want to highlight that we applied the postprocessing steps, such as morphological region filling and blob removal, to enhance the final segmentation results. In Fig. 2, the first column showcases the exemplar OCT scans taken from different OCT datasets reported in Table 1. The second column showcases the ground truths, which we have added here for visual evaluation purposes only. The third column showcases the proposed model’s results; the fourth column depicts Swin-UNETR’s^53^ results; the fifth column showcases RLSRC’s^55^ results, and the sixth column showcases the HyFormer’s^54^ results. From Fig. 2, we can observe that the proposed model was able to achieve promising results toward extracting the retinal lesions as compared to the SOTA models. Similarly, due to the multiscale decomposition mechanism injected within the proposed network, it segmented concealed lesions, e.g., see the extracted HE in Figure 2 (I and O) compared to the ground truths shown in Fig. 2 (H and N), respectively. Furthermore, thanks to the IB, SPB, HDB, and Hardswish-based transformer blocks within the proposed model, the proposed model was able to extract the retinal lesions while retaining their shape information. For example, notice the extracted IRF, SRF, Drusen, and CNV in Fig. 2 (C, U, AA) compared to the ground truths shown in Fig. 2 (B, N, and Z), respectively. In Fig. 3, we can notice that although the proposed system was able to extract the retinal lesions, it was not able to preserve the shape of the lesions very accurately compared to the ground truth annotations. This segmentation gap was expected because the proposed system was trained only once using the text prompts rather than pixel-level ground truth annotations, and applied to all the datasets, reported in Table 1, to extract retinal lesions. But considering the fact that the proposed system was able to extract all the retinal lesions without requiring exhaustive re-training or fine-tuning rounds, the segmentation results produced by the proposed system are appreciable. Moreover, the ability of the proposed system to infer learning from the text prompts rather than pixel-level ground truth annotations highlights its deployment potential in the real-world hospital setting to segment retinal lesions robustly.

**Table 7:** Performance comparison of the proposed model with SOTA methods in terms of computational complexity and execution time. The best scores are in bold, while the second-best scores are in italics.

Model	GFLOPS	Time (ms)
Proposed	**6.24**	**10.83**
Swin-UNETR^53^	12.8	16.76
HyFormer^54^	*7.97*	*12.20*
RLSRC^55^	16.14	20.54
RAG-FW^9^	34.08	48.36

### Limitations

Although the proposed model exhibited a good performance toward extracting retinal lesions from the OCT images of six public datasets, we observed a few cases where the proposed system’s performance was a bit limited. For instance, in Fig. 2O, the proposed model missed the presence of tiny HE regions, which can be observed in the clinician’s annotations in Fig. 2N. Similarly, the presence of a tiny IRF region was missed by the proposed model in Fig. 2I as compared to the clinician’s annotations reported in Fig. 2H. Apart from this, the proposed model was a bit limited in preserving the thickness of the extracted Drusen in Fig. 2U as compared to the clinician’s annotations reported in Fig. 2T. Moreover, in Fig. 3, we can observe that the proposed system managed to recognize the retinal lesions, but was not able to preserve their shapes well as compared to the ground truth. Despite these limitations, the proposed model was able to produce better lesion extraction performance as compared to the SOTA models (see Fig. 2). But these limitations can be eliminated in the future by increasing the depth of the multiscale decomposition within the proposed model to extract even the smallest lesions which were missed by the proposed model in Fig. 2I, O. But observing the magnitude of the performance improvements over the increase in the computational complexity, which we will get in addressing these limitations, we did not pursue these remedies in this work.

**Table 8:** Performance of the proposed framework in terms of mean intersection-over-union across each dataset after multiple runs.

Dataset	Performance (Mean ± STD)
BIOMISA^45^	0.9427 ± 0.0086
Zhang^29^	0.9036 ± 0.0015
Duke-1^46^	0.9283 ± 0.0031
Duke-2^17^	0.9363 ± 0.0049
Duke-3^47^	0.9216 ± 0.0042
Rabbani^48^	0.9129 ± 0.0062

## Discussion

The paper proposes a novel language-assisted multimodal convolutional transformer pipeline that can segment retinal lesions from the OCT images by aligning its image features with the clinically significant text features obtained from the fine-tuned text encoder using the $L_s$ loss function. Unlike the conventional deep learning models, which require hefty pixel-level annotations for learning the lesions extraction tasks, the proposed model relies on text representations derived from the clinical text prompts to learn the lesions segmentation task. This ability of the proposed model to infer learning from the text prompts makes it highly generalizable, and it can adapt to any OCT dataset to extract the retinal lesions with only one-time training. We also wanted to clarify that the segmentation process within the proposed pipeline is conventional, where in the first step, the model generates latent space representations from the candidate input scan, and in the second step, the model utilizes the generated latent space representations in order to compute the output segmentation masks. In fact, the proposed scheme can be applied to any conventional segmentation model, as evidenced by the ablation study reported in Table 4 and Section 6.1. However, it should be noted that in contrast to how the latent space representations are refined by updating the weights of the model using supervised ground truth labels in the conventional models, we propose the use of image-text alignments (in the latent space) using the proposed loss function, where the image features (generated by the image/vision encoder) are refined based on the text features produced from the pretrained text encoder. So, instead of relying on the pixel-level ground truth annotations, the proposed scheme allows the underlying segmentation model to learn from the text features produced from the pretrained language model. Additionally, we have also tested the proposed system on each dataset across ten runs and measured its performance using mIoU. Then, we took the average of mIoU scores across ten runs and also computed the standard deviation to analyze the variability in the proposed system’s performance. The results are reported in Table 8 from which we can observe that for each dataset, the variability of the proposed system is minimal, and ir produced a consistent and highly reproducible retinal lesion segmentation performance. Apart from this, many SOTA models focus on extracting low-level and high-level visual cues from OCT images^9,54,55^. However, these features may not always correspond to the actual clinical manifestations of retinal lesions. By incorporating domain knowledge in the form of textual prompts, the proposed model increases interpretability and clinical relevance, yielding more accurate lesion segmentation. Furthermore, the proposed model provides the best trade-off between segmentation performance, computational complexity, and run-time performance as compared to the SOTA models (see Tables 6, and 7 for more details). Although there were a few cases, as discussed in “Limitations”, in which the performance of the proposed model was a bit limited. However, these cases can be remedied easily in the future. Also, despite these limitations, the proposed model was able to produce better lesion segmentation results than the SOTA models. Apart from this, the proposed model was tested on six public datasets, on which it consistently outperformed the SOTA works in terms of mIoU, mDC, and $\mu _{map}$ scores. Additionally, due to its ability to adapt to different datasets with one-time training, it can be a highly scalable model to be deployed in the real-world hospital setting to assist doctors in segmenting the retinal lesions from the DME and AMD-affected scans. Similarly, the proposed scheme provides a clinically interpretable, annotation-free, and dataset-agnostic approach for retinal lesion segmentation. The integration of language-assisted learning, multiscale feature extraction, and a robust one-time training mechanism allows the proposed scheme to overcome the limitations of existing SOTA methods and establish a new benchmark for retinal lesion segmentation using OCT scans.

In the future, we plan to extend the applicability of the proposed scheme to other medical applications for extracting different lesions and biomarker information.

## Conclusion

This paper proposes a novel language-driven multimodal convolutional transformer pipeline for extracting retinal lesions from OCT scans. Unlike the conventional models, which require extensive pixel-level ground truths to learn the lesions segmentation task accurately, the proposed model relies on the text features derived from the clinical text prompts to learn the lesions segmentation task. This ability of the proposed model to infer learning from the text prompts rather than pixel-level ground truth annotations allows it to adapt to any OCT dataset to extract the retinal lesions with only one-time training. Apart from this, it makes the proposed model intrinsically scalable, as it eliminates the need for re-training or fine-tuning the model again to adapt to different datasets for performing the retinal lesions extraction task. The proposed model was rigorously evaluated on six public datasets where it consistently outperformed the SOTA approaches by achieving up to 7.77%, 5.70%, and 4.21% better results in terms of mIoU, $\mu _{map}$, and mDC scores, respectively. In the future, we envisage testing the proposed scheme in other medical applications to extract different lesion pathologies and biomarker information.

## Data Availability

All the data used in this research is publicly available. The link to each of the datasets is as follows: (1) BIOMISA, Link: https://data.mendeley.com/datasets/trghs22fpg/1/ (2) Duke-1, Link: https://people.duke.edu/ sf59/RPEDC_Ophth_2013_dataset.htm (3) Duke-2, Link: https://people.duke.edu/ sf59/Chiu_BOE_2014_dataset.htm (4) Duke-3, Link: https://people.duke.edu/ sf59/Srinivasan_BOE_2014_dataset.htm (5) Rabbani, Link: https://tinyurl.com/bdf2hnhb (6) Zhang, Link: https://data.mendeley.com/datasets/rscbjbr9sj/3
